# The Identification and Characterization of WOX Family Genes in *Coffea arabica* Reveals Their Potential Roles in Somatic Embryogenesis and the Cold-Stress Response

**DOI:** 10.3390/ijms252313031

**Published:** 2024-12-04

**Authors:** Xiangshu Dong, Jing Gao, Meng Jiang, Yuan Tao, Xingbo Chen, Xiaoshuang Yang, Linglin Wang, Dandan Jiang, Ziwei Xiao, Xuehui Bai, Feifei He

**Affiliations:** 1 School of Agriculture, Yunnan University, Kunming 650500, China; dongxiangshu@ynu.edu.cn (X.D.); jinggao@ynu.edu.cn (J.G.); jiangmeng_xo9c@stu.ynu.edu.cn (M.J.); taoyuan_orz3@stu.ynu.edu.cn (Y.T.); chenxingbo@mail.ynu.edu.cn (X.C.); yangxiaoshuang@mail.ynu.edu.cn (X.Y.); wanglinglin@ynu.edu.cn (L.W.); dandanjiang32@163.com (D.J.); 2 Dehong Tropical Agriculture Research Institute, Dehong 678600, China; xzw464999488@126.com (Z.X.); 13529520059@163.com (X.B.)

**Keywords:** *Coffea arabica*, WOX gene, somatic embryogenesis, cold stress

## Abstract

*WUSCHEL-related homeobox* (*WOX*) genes play significant roles in plant development and stress responses. Difficulties in somatic embryogenesis are a significant constraint on the uniform seedling production and genetic modification of *Coffea arabica*, hindering efforts to improve coffee production in Yunnan, China. This study comprehensively analyzed *WOX* genes in three *Coffea* species. A total of 23 *CaWOXs*, 12 *CcWOXs*, and 10 *CeWOXs* were identified. Transcriptomic profile analysis indicated that about half of the *CaWOX* genes were actively expressed during somatic embryogenesis. The most represented *CaWOXs* were *CaWOX2a*, *CaWOX2b*, *CaWOX8a*, and *CaWOX8b*, which are suggested to promote the induction and development of the embryogenic callus, whereas *CaWOX13a* and *CaWOX13b* are suggested to negatively impact these processes. Co-expression analysis revealed that somatic embryogenesis-related *CaWOXs* were co-expressed with genes involved in embryo development, post-embryonic development, DNA repair, DNA metabolism, phenylpropanoid metabolism, secondary metabolite biosynthesis, and several epigenetic pathways. In addition, qRT-PCR showed that four *WOX* genes responded to cold stress. Overall, this study offers valuable insights into the functions of *CaWOX* genes during somatic embryogenesis and under cold stress. The results suggest that certain *WOX* genes play distinct regulatory roles during somatic embryogenesis, meriting further functional investigation. Moreover, the cold-responsive genes identified here are promising candidates for further molecular analysis to assess their potential to enhance cold tolerance.

## 1. Introduction

WUSCHEL-related homeobox (WOX) proteins are named in reference to the *Arabidopsis* stem cell regulator, WUSCHEL, and belong to a plant-specific subgroup of the homeodomain (HD) superfamily [1,2,3]. The homeodomain of HD superfamily proteins comprises 60 amino acid residues that arrange themselves into a distinct “helix–loop–helix–turn–helix” three-dimensional configuration [4]. The second and third helical segments of the domain unite to form a “helix–turn–helix” motif, facilitating precise recognition and binding to specific DNA sequences [4,5]. Compared to other members of the HD superfamily, WOX proteins exhibit unique additional motifs, including FYWFQNH, FYWFQNR, and YNWFQNR, which correlate specifically with their phylogenetic groups (the WUS/modern, intermediate, and ancient clades, respectively) [2,6]. The ancient clade encompasses conserved *WOX* genes across diverse taxa, from algae to angiosperms, while the intermediate clade comprises members in plant groups like ferns to angiosperms. Lastly, members of the WUS/modern clade are exclusively present in seed plants [7].

Previous studies have demonstrated that WOX genes play diverse functional roles during plant developmental processes, including embryonic patterning, stem cell maintenance, flower architecture, and somatic embryogenesis [8,9]. For example, *AtWUS* defines stem cell and floral meristem identities, while also regulating maintenance of the shoot apical meristem (SAM) [1,10]. Overexpression of *WUS* genes in many plants, such as *Arabidopsis*, *Gossypium hirsutum*, and *Coffea canephora*, can increase their ability to form somatic embryos [11,12,13,14]. Numerous *AtWOX* genes and their corresponding functions have been characterized in Arabidopsis, providing an important framework for understanding their roles in plant development. *AtWOX1* plays a pivotal role in meristem development through the regulation of *CLV3* expression and *SAMDC* activity [15]. During embryo development, *AtWOX8* and *AtWOX9* redundantly affect basal and apical lineage development by influencing the expression of *AtWOX2* and localizing the auxin response [16]. Notably, *AtWOX2* serves as a marker for the apical cell lineage and is highly expressed in the egg cell and zygote, independent of exogenous auxin influence [16]. *AtWOX3* is expressed in leaf and floral primordia margins, recruiting founder cells from meristem layers to form lateral domains in vegetative and floral organs [17]. Research has shown that *AtWOX4* and *AtWOX14* act redundantly to promote the proliferation and differentiation of procambial cells via distinct pathways. In particular, *AtWOX4* interacts with *PXY* (*Phloem intercalated with xylem*) to augment auxin responsiveness in the cambium [18]. Conversely, *AtWOX14* promotes vascular cell differentiation and lignification in the inflorescence stems of *Arabidopsis* by increasing the accumulation of bioactive gibberellin (GA) [19,20]. Under the control of *Auxin Response Factor 10* (*ARF10*) and *ARF16*, *AtWOX5* is expressed in the quiescent center (QC), functioning as a local, QC-specific regulator that activates *TAA1*-mediated auxin biosynthesis to regulate the functioning of the distal meristem [21,22]. During ovule development, *AtWOX6* is expressed in the embryo, suspensor, and endosperm nuclei but is absent in the integument [23]. *AtWOX7*, which is activated by sugar and repressed by auxin, regulates lateral root development through the direct inhibition of the cell cycle gene *CYCD6;1* [24]. *CYCD6;1* can also be independently induced by auxin during asymmetric stem cell division in the root [25]. *AtWOX11* serves as a direct target of the auxin signaling pathway, regulating founder cell establishment during de novo root regeneration and callus formation [26]. *AtWOX12* acts redundantly with *AtWOX11* and is involved in the first step of the cell fate transition during de novo root organogenesis in *Arabidopsis* [27]. Lastly, *AtWOX13* is induced by auxin and regulates the cell fate determination of pluripotent callus to inhibit new shoot generation [28]. Thus, *WOX* genes contribute diverse roles to several developmental processes in *Arabidopsis* through their interactions with auxin and other homomers.

In addition to their roles in plant and somatic embryogenesis development, *WOX* genes are also involved in responses to abiotic stressors in many plant species, including cold, drought, and salt stress [29,30]. In *Arabidopsis*, *AtWOX6* plays a role in freezing tolerance by affecting the activities of gene products that are independent of the C-repeat binding factor (CBF) pathway [31]. In addition to *Arabidopsis*, many *WOX* genes have been identified that respond to cold stress and other abiotic stressors in rice, pineapple (*Ananas comosus* L.), paper mulberry, soybean, and tea tree (*Camellia sinensis*) plants [32,33,34,35,36].

*Coffea arabica* L., known as Arabica coffee, belongs to the Rubiaceae family and accounts for over 70% of coffee bean production [37]. In China, over 98% of the cultivation and yield of *C. arabica* originates from the Yunnan province [38]. With the increase in demand for Arabica coffee beans, *C*. *arabica* cultivation has spread towards the highlands in Yunnan, where the temperature occasionally reaches 0 °C [39,40]. Thus, cold tolerance is a desired characteristic of *C. arabica* plants in this region, in addition to uniform clonal seedlings, since the yield and quality of traditional seedlings produced by seeds are unstable due to the allotetraploid (2n = 4x = 44 chromosomes) and autogamous nature of *C. arabica*. Therefore, understanding cold-responsive and somatic embryogenesis developmental mechanisms in *C. arabica* can assist in the development of cold-tolerant varieties, improve the efficiency of somatic embryogenesis, and shorten the somatic embryogenesis period.

The aim of the current study was to systematically identify WOX genes in *C. arabica* based on newly published genome data [41] and to reveal the cold-responsive and somatic embryogenesis developmental roles of *Coffea arabica WUSCHEL-related homeobox* (*CaWOX*) genes. Our results enhance our understanding of the roles of the *CaWOX* gene family in cold responses and somatic embryogenesis, thus potentially accelerating the molecular breeding programs for *Coffea* spp. plants in China.

## 2. Results

### 2.1. Identification and Phylogenetic Analysis of WOX Genes in Three Coffee Plants

**Figure 1:**
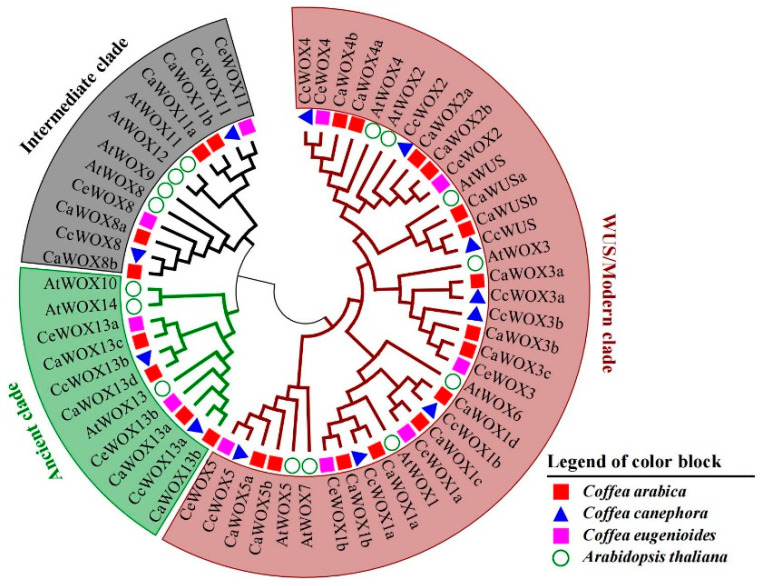
Phylogenetic relationships of WOX proteins from three coffee plants and *Arabidopsis*. Different clades are indicated by the background colors and branch lines.

Based on HMM and BLASTp searches, 23 CaWOXs, 12 CcWOXs, and 10 CeWOXs were identified in the genomes of *C. arabica*, *C. canephora*, and *C. eugenioides,* respectively (Table 1). The identified CaWOXs/CcWOXs/CeWOXs were named based on their best matching homologs in *Arabidopsis*. The number of WOXs identified in tetraploid *C. arabica* was comparable to the combined total found in its diploid progenitors, *C. canephora* and *C. eugenioides*. Similar to previous results from *Arabidopsis*, phylogenetic analysis suggested that the WOX proteins from *Arabidopsis* and coffee plants belonged to three clades including the WUS/modern, ancient, and intermediate-aged clades (Figure 1). The WUS clade was the largest group in this phylogenetic tree, containing 15, 8, 7, and 8 members from *C. arabica*, *C. canephora*, *C. eugenioides*, and *Arabidopsis*, respectively. Compared with *AtWOXs*, some *WOX* gene families, such as *WOX1* and *WOX13*, were expanded in the three coffee plants, while *WOX3* family expansion was only observed in *C. arabica* and *C. canephora* (Figure 1). *WOX6*, *WOX7*, *WOX9*, *WOX10*, *WOX12*, and *WOX14* were not encoded by the three coffee plant genomes (Figure 1), potentially due to the whole-genome polyploidization event that occurred in the lineage leading to *Arabidopsis*, which diverges from the common ancestor shared with *Coffea* [42].

### 2.2. Gene Structure and Domain Analysis of CaWOXs

**Figure 2:**
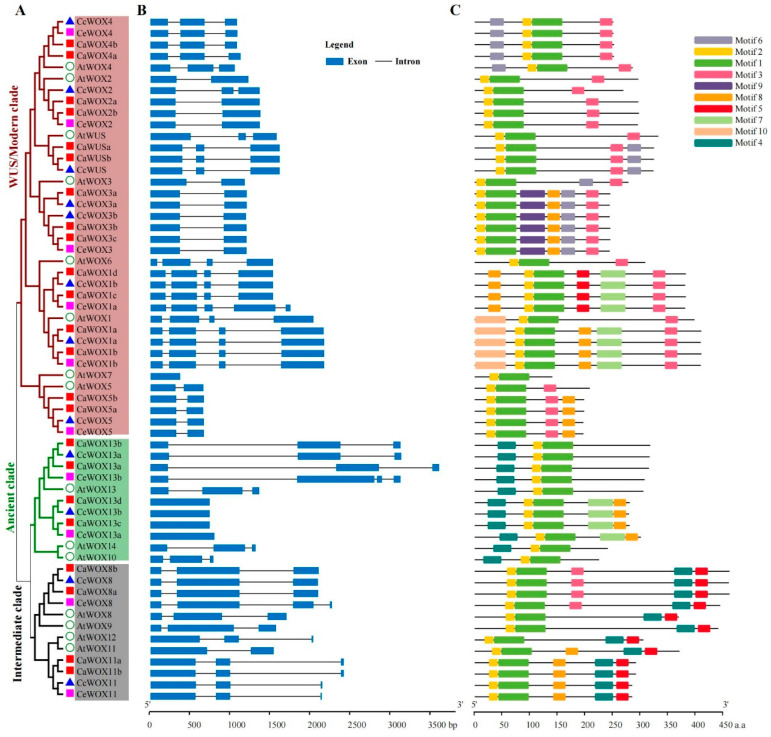
Phylogenetic reconstruction (**A**), gene structures (**B**), and MEME motifs (**C**) for WOXs identified in three coffee plants and Arabidopsis. (**B**) Dark blue boxes represent exons and black lines indicate introns. (**C**) Colored boxes indicate motifs, as shown in the legend on the right.

To explore *WOX* diversity, their gene structures and conserved motifs were analyzed. Most *WOX* genes, except *WOX13* and *WOX7*, contained at least one intron, and two introns represented the most common gene structure configuration (Figure 2B). Members from the same *WOX* gene family exhibited similar gene structures, indicating that they potentially encoded conserved functions. Analysis of the conserved motifs indicated that 10 were identified in WOXs (Figure 2C). The 10 motifs of the typical WOX proteins followed the order of Motif 6–Motif 2–Motif 1–Motif 3–Motif 9–Motif 8–Motif 5–Motif 7–Motif 10–Motif 4. Motif 1 contained a homeodomain conserved sequence, but the other motifs did not share significantly conserved motifs based on BLAST searches in the National Center for Biotechnology Information (NCBI) and protein families (Pfam) databases (Figure 2C and Table S1). Furthermore, Motif 1 was found in all WOXs, while Motif 4 was only found in the ancient and intermediate clades, with no annotation target from the public protein database. Motif 9 was only found in Ca/Cc/CeWOX3 (Figure 2C and Table S1).

### 2.3. Expression Profiles of CaWOXs in Different Tissues

**Figure 3:**
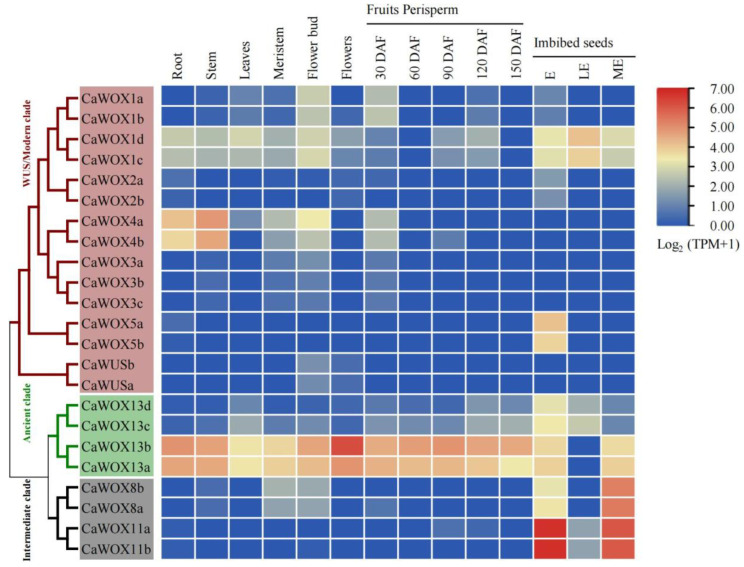
Expression of *CaWOX* genes in different tissues and organs of *Coffea arabica* based on RNA-sequencing data. Expression data were treated with log_2_(TPM+1) normalization, as indicated by the scale to the right. DAF, day after flowering. E, seed embryo. LE, seed lateral endosperm. ME, seed micropylar endosperm.

To evaluate the functions of *CaWOXs* during development, their expression patterns were compared using RNA-sequencing (RNA-seq) data from 14 organs or tissues, including roots, stems, leaves, meristems, flower buds, flowers, fruit perisperm (five samples), and 10-day imbibed seeds (three samples) (Table S2). *CaWOXs* were differentially expressed among the 14 tissue samples (Figure 3). *CaWOX4a* and *CaWOX4b* were predominantly expressed in roots and stems. *CaWOX1c* and *CaWOX1d* were mostly expressed in all tissues of 10-day imbibed seeds. *CaWOX5a*, *CaWOX5b*, *CaWOX13c*, and *CaWOX13d* were predominantly expressed in embryos of 10-day imbibed seeds. *CaWOX8a*, *CaWOX8b*, *CaWOX11a*, and *CaWOX11b* were mainly expressed in the embryo and micropylar endosperm of 10-day imbibed seeds. Furthermore, *CaWOX13a* and *CaWOX13b* were predominantly expressed in all tissues except the lateral endosperm of 10-day imbibed seeds (Figure 3).

### 2.4. Expression Patterns of CaWOX Genes During Somatic Embryogenesis

**Figure 4:**
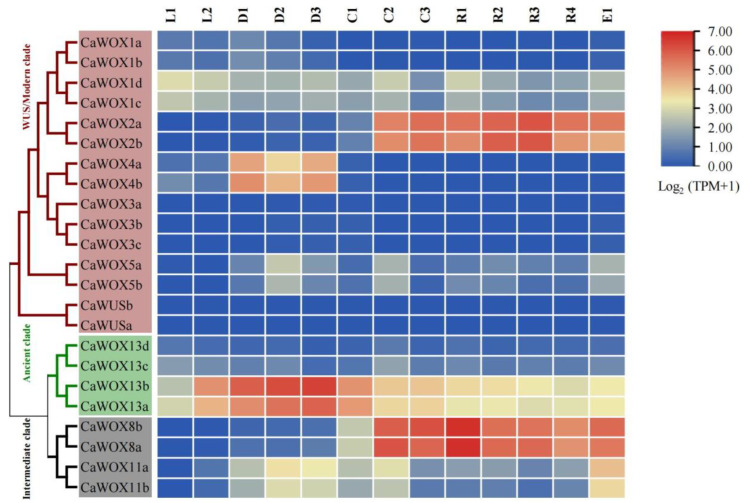
Expression of *CaWOX* genes during somatic embryogenesis in *Coffea arabica* based on RNA-sequencing data. Expression data were treated with log_2_(TPM + 1) normalization, as indicated by the scale to the right. L1, leaves from plants. L2, D1, D2, and D3, leaf explants during dedifferentiation after 0 h, 1 week, 2 weeks, and 5 weeks, respectively. C1, primary callus. C2, embryogenic callus. C3, cell clusters obtained from proliferation medium. R1, cell clusters after induction in DIF (redifferentiation) medium for 1 week. R2, R3, and R4, cell clusters after 24 h, 72 h, and 10 d of reducing cell density, respectively. E1, globular embryos. E2, torpedo-shaped embryos.

**Figure 5:**
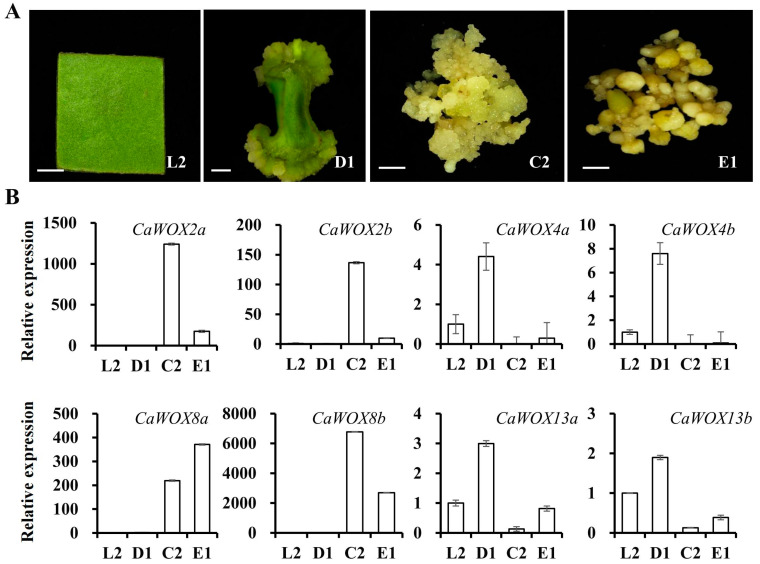
Expression patterns of *CaWOX* genes related to somatic embryogenesis. (**A**), four key stages selected for qRT-PCR analysis. L2 and D1, leaf explants during dedifferentiation after 0 h and 1 week, respectively. C2, embryogenic callus. E1, globular embryos. Bar = 2 mm. (**B**), qRT-PCR results of *CaWOXs* related to somatic embryogenesis within four key stages. Expression levels were represented with the method of 2^−∆∆CT^.

Previous research has reported that *WOX* genes play key roles during somatic embryogenesis [14]. To explore the functions of *CaWOXs* during somatic embryogenesis, the *CaWOX* expression patterns from leaf explants (L1) to globular embryos (E1) during 13 key developmental stages were re-calculated and re-analyzed from previous studies (Figure 4 and Table S3) [43]. The expression levels of *CaWOX4a* and *CaWOX4b* were mainly expressed from 1 to 5 weeks of explant dedifferentiation (D1–D3). *CaWOX13a* and *CaWOX13b* were highly expressed from leaf explants (L2) to primary callus (C1), while *CaWOX2a*, *CaWOX2b*, *CaWOX8a*, and *CaWOX8b* were predominantly expressed from embryogenic callus (C2) to globular embryos (E1). Further, the expression levels of these eight *CaWOXs* were analyzed again with four samples (L2, D1, C2, and E1) during the 13 key developmental stages using qRT-PCR (Figure 5). Based on qRT-PCR results, *CaWOX2a*, *CaWOX2b*, *CaWOX8a*, and *CaWOX8b* were highly expressed in C2 and E1 samples, while *CaWOX4a*, *CaWOX4b*, *CaWOX13a*, and *CaWOX13b* were highly expressed in D1 samples (Figure 5B and Table S4).

**Figure 6:**
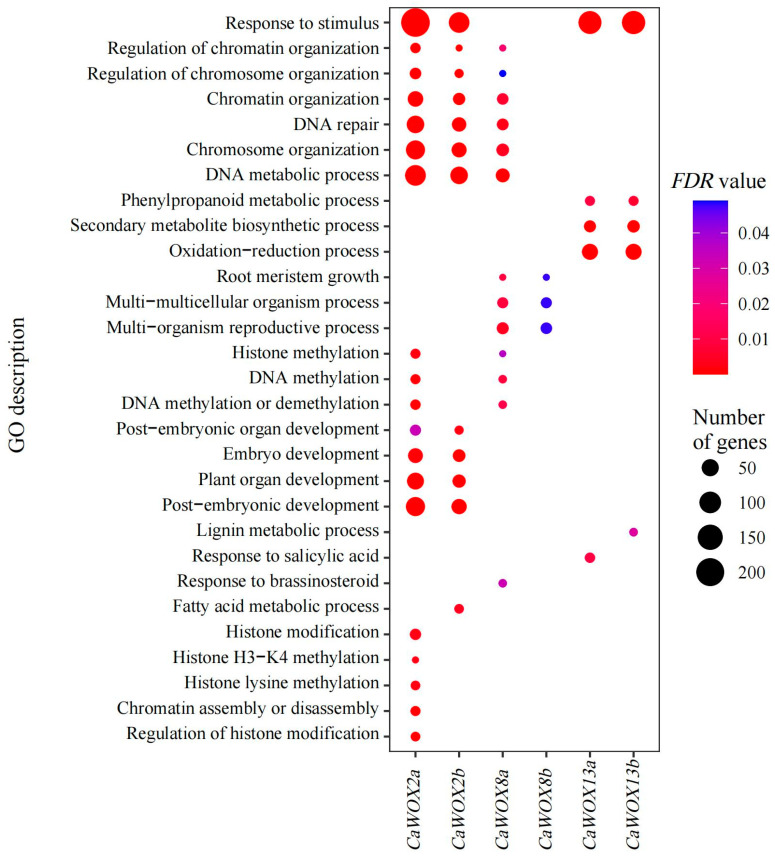
Gene Ontology (GO) enrichment analysis of genes co-expressed with *CaWOX2a*, *CaWOX2b*, *CaWOX8a*, *CaWOX8b*, *CaWOX13a*, and *CaWOX13b* which involved in somatic embryogenesis. FDR, false discovery rate.

These results suggest that *CaWOX2a*, *CaWOX2b*, *CaWOX8a*, and *CaWOX8b* promote the induction and development of the embryogenic callus, whereas *CaWOX13a* and *CaWOX13b* may have negative impacts on this process. To further identify the functions of *CaWOX2a*, *CaWOX2b*, *CaWOX8a*, *CaWOX8b*, *CaWOX13a*, and *CaWOX13b* during embryogenic callus induction and development, co-expression analysis was carried out using the RNA-seq datasets. Considering a PCC value of PCC < −0.9 or >0.9 as reflecting a strong correlation, *CaWOX2a*, *CaWOX2b*, *CaWOX8a*, *CaWOX8b*, *CaWOX13a*, and *CaWOX13b* were co-expressed with 1052, 407, 520, 500, 558, and 536 genes, respectively (Table S5). Subsequently, GO enrichment analysis was carried out to identify the biological processes related to these co-expressed genes (Figure 6). Embryo development, plant organ development, post-embryonic development, and post-embryonic organ development were represented by genes co-expressed with *CaWOX2a* and *CaWOX2b* (Figure 6). Root meristem growth, multicellular organismal process, and multi-organism reproductive process were among the categories of genes co-expressed with *CaWOX8a* and *CaWOX8b* (Figure 6). In addition, phenylpropanoid metabolic, secondary metabolite biosynthetic, and oxidation–reduction processes were represented by genes co-expressed with *CaWOX13a* and *CaWOX13b* (Figure 6). Furthermore, regulation of chromatin organization, regulation of chromosome organization, DNA repair, and DNA metabolic process were represented by genes co-expressed with *CaWOX2a*, *CaWOX2b*, and *CaWOX8a* (Figure 6). Histone modification, histone H3-K4 methylation, histone lysine methylation, chromatin assembly or disassembly, and the regulation of histone modification were functions related to the genes co-expressed with *CaWOX2a* (Figure 6).

### 2.5. Expression Patterns of CaWOX Genes Under Cold Stress and Other Stress Treatments

**Figure 7:**
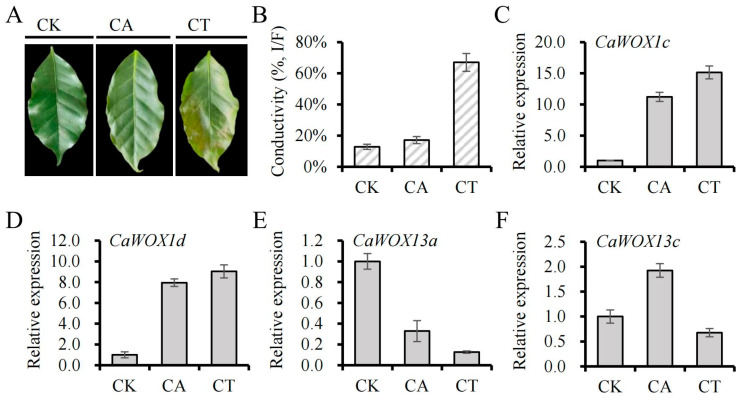
Phenotypic analysis of *Coffea arabica* seedlings after cold treatment and the relative expression patterns of *CaWOX* genes after cold treatment. (**A**), phenotype of *Coffea arabica* leaves after cold treatment. (**B**), electrolyte leakage of *Coffea arabica* leaves after cold treatment. (**C**–**F**), relative expression levels of cold-responsive *CaWOXs* based on qRT-PCR results. CK, control condition, the *C. arabica* seedlings were grown at 24/20 °C (day/night). CA, cold acclimatization, seedlings were grown at 13/8 °C (day/night). CT, cold treatment, seedlings were exposed to 4/4 °C (day/night).

Cold stress can be a major natural disaster for Chinese coffee production. *WOX* genes have been reported to be responsive to cold stress [32,33]. The expression patterns of *CaWOXs* under cold stress were consequently analyzed with qRT-PCR using the two top pairs of mature leaves of one-year old *C. arabica* seedlings subjected to cold treatment. After cold acclimatization (CA), the tips of *C. arabica* leaves exhibited symptoms of frost damage, and under cold treatment (CT), these symptoms spread to cover half of the leaves (Figure 7A). In agreement with the symptoms observed on the leaves, electrical conductivity increased minimally under CA conditions, but increased by up to 67% under CT conditions (Figure 7B). The expressions of four *CaWOXs* were identified as responsive to cold treatment when considering thresholds of a fold-change exceeding two (Figure 7C–F). Specifically, *CaWOX1c* and *CaWOX1d* expression increased under both CA and CT conditions, whereas *CaWOX13a* expression decreased under both CA and CT conditions (Figure 7C–E). *CaWOX13c* initially demonstrated increased expression under CA conditions but subsequently returned to normal expression levels under CT conditions (Figure 7E,F).

**Figure 8:**
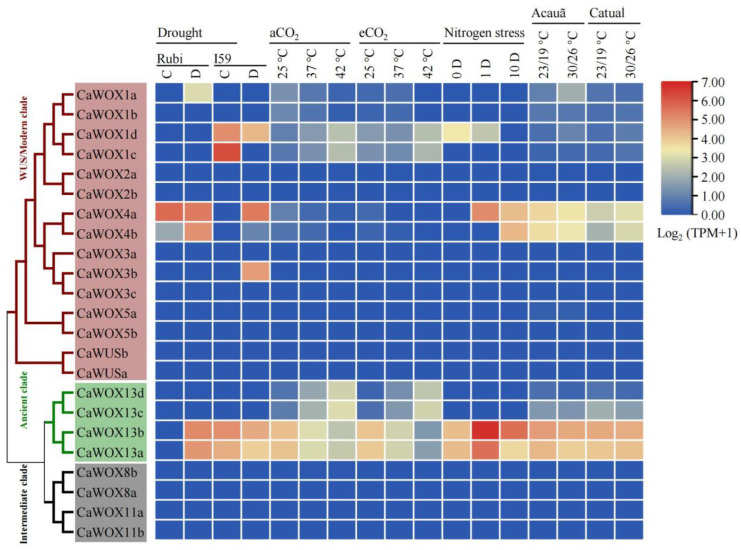
Expression patterns of CaWOX genes under different stress treatments based on RNA-sequencing data. Rubi, drought-susceptible *C. arabica* cultivar Rubi MG1192. I59, drought-tolerant *C. arabica* cultivar IAPAR59. C, control. D, drought treatment. aCO_2_, ambient air CO_2_ (380 µL L^−1^). eCO_2_, elevated air CO_2_ (700 µL L^−1^). The terms 0 D, 1 D, and 10 D indicate 0, 1, and 10 days after nitrogen starvation. Acauã, *C. arabica* cultivar Acauã. Catuaí, *C. arabica* cultivar Catuaí IAC 144.

In addition to cold stress, drought, elevated atmospheric CO_2_, and high-temperature stress are also considered among the most impactful stressors in global coffee production. To further evaluate the functions of *CaWOXs* of *C. arabica* in stress response, RNA-seq data for *CaWOXs* under drought stress, elevated temperatures stress, elevated air CO_2_ stress, and nitrogen stress were re-evaluated from previous studies [44,45,46]. Briefly, *CaWOX1d* exhibited higher expression in the drought-tolerant *C. arabica* cultivar IAPAR59 (I59) under both control and drought treatments (Figure 8). *CaWOX3b* was only expressed in the drought-tolerant cultivar (I59) after drought treatment, while *CaWOX4b* exhibited a response to drought only in the drought-susceptible cultivar (Rubi) (Figure 8). When exposed to elevated air CO_2_, *CaWOX* expression was not significantly different. However, *CaWOX1c*, *CaWOX1d*, *CaWOX13c*, and *CaWOX13d* exhibited weak expression responses to elevated temperatures under both ambient and elevated air CO_2_ concentrations (Figure 8). Under nitrogen stress, both *CaWOX4a* and *CaWOX4b* exhibited increased expression, but CaWOX4b displayed a lagged response (Figure 8). In addition, *CaWOX4a* and *CaWOX4b* were more highly expressed in the heat-sensitive cultivar Catuaí under both control and water conditions, in addition to being continuously expressed in the heat-tolerant cultivar Acauã under these conditions (Figure 8).

## 3. Discussion

### 3.1. WOX Genes in C. arabica

WOX genes are important in plant development, and thus, genome-wide analyses of the WOX gene family have been conducted in *Arabidopsis*, rice, cucumber, soybean, *Rosa hybrida*, *Nelumbo nucifera*, *Medicago sativa*, and tea (*Camellia sinensis*) plants [6,9,35,36,47,48,49]. In the present study, 23 *WOX* genes were identified in *C. arabica*, in addition to 12 members in *C. canephora* and 10 in *C. eugenioides* (Table 1). Previously, only seven members had been identified in *C. arabica* based on an in silico analysis of the expressed sequence tag database [50]. The number of WOXs isolated in the current study was significantly higher, owing to analysis of a newly published chromosome-level genome for *C. arabica* [41], leading to greater accuracy in *CaWOX* identification. *Arabidopsis* underwent genome polyploidization and gene loss events, resulting in a greater number of *AtWOX* genes compared to the number of *CcWOXs* and *CeWOXs* identified in *Coffea* plants (Table 1) [42,51].

### 3.2. CaWOX Functions

The evaluation of gene expression patterns can help to predict gene functions. Consequently, *CaWOX* gene expression profiles were analyzed from previously published datasets from 14 diverse tissues, in addition to expressional profiles responsive to drought, elevated atmospheric CO_2_, and high-temperature stress (Figure 3 and Figure 8). Some *CaWOX* genes exhibited tissue-specific expression, while others exhibited differential expression in response to drought, elevated atmospheric CO_2_ levels, and high temperatures. For example, homologs of *AtWOX4*, *CaWOX4a*, and *CaWOX4b* were predominantly expressed in roots and stems (Figure 3). In *Arabidopsis*, *AtWOX4* confers auxin responsiveness to cambium cells and is responsible for extended root and stem thickening [18]. These results indicate that the function of *WOX4* may be conserved among species and that CaWOX4 genes may confer functional redundancy. Furthermore, *AtWOX11* has been reported to play a role during the induction of seed dormancy and release stages [52]. The high expression levels of *CaWOX11a* and *CaWOX11b* in the seed embryo and seed micropylar endosperm of imbibed seeds suggests similar functioning (Figure 3). These results collectively offer new insights for future functional prediction and characterization of *CaWOXs*.

### 3.3. CaWOX and Somatic Embryogenesis

Somatic embryogenesis (SE) is a micropropagation technique through which plants can regenerate bipolar structures from somatic cells, thus producing genetically uniform seedlings. In China, achieving embryonic competence in callus tissue remains a formidable challenge in the process of somatic embryogenesis for *C. arabica*, thereby impeding the progress of the coffee industry. Understanding the genes that respond during the SE process can offer valuable insights for accelerating it [53]. Previous studies have demonstrated that the overexpression of *WOX* genes, such as *AtWUS*, *TaWOX5*, and *ZmWUS2*, can induce somatic embryogenesis-related genes and promote spontaneous regeneration [54,55,56]. In *C. arabica*, a *WOX-like* gene belonging to the intermediate clade has been shown to be related to the embryogenic process [50], while other members remain unstudied. In this study, comprehensive investigation and characterization of *CaWOXs* in somatic embryogenesis was conducted via genome-wide analysis. Further, the expression levels of highly related genes were confirmed using samples from key stages, ranging from explants to torpedo-shaped embryos (Figure 4 and Figure 5). Unlike previous studies in *Arabidopsis* and maize [55,57], *CaWUS* was not expressed during *C. arabica* somatic embryogenesis, while *CaWOX2s* and *CaWOX8s* were markedly increased in expressed during the transformation from primary (non-embryonic callus) to embryonic callus, indicating that these genes play vital roles in the key embryonic transition process (Figure 4). In cotton, the *WOX8* and *WOX2* genes play similar roles instead of *WUS*, or they merely function in a redundant manner [14].

Considering the roles of *WOX2* and *WOX8* during somatic embryogenesis, a previous study has shown that *PpWOX2* (*Pinus pinaster WOX2*, a homolog of *AtWOX2*) promotes somatic embryogenesis and organogenesis in *Arabidopsis* [58]. In *Arabidopsis*, *WOX8* and *WOX9* are expressed at very early stages of embryo development, and ectopic co-expression of *WOX2* with either *WOX8* or *WOX9* enhances regeneration from leaf segments and free cells in *Nicotiana tabacum*, suggesting that they may be critical regulators of somatic embryogenesis [59,60]. In woody plants, the expression patterns of *WOX2*, *WOX8*, and *WOX9* indicate their involvement in regulating somatic embryogenesis [61]. In *C. arabica*, no orthologs of *AtWOX9* were identified. However, *CaWOX2s* and *CaWOX8s* exhibited high expression levels from the embryogenic callus stage to the torpedo-shaped embryo stage, indicating their roles as crucial regulators of somatic embryonic properties (Figure 4).

In addition to *CaWOX2s* and *CaWOX8s*, *CaWOX13a* and *CaWOX13b* were responsive to the transformation process from non-embryonic to embryonic callus, but they exhibited decreased expression during this stage (Figure 4). In *Arabidopsis*, *AtWOX13* suppresses de novo shoot regeneration from callus and impacts regeneration efficiency in *Arabidopsis* [28], suggesting that *AtWOX13* and its homologs in other plants play negative roles during the key embryonic transition process.

### 3.4. Potential Mechanisms of CaWOXs in Somatic Embryogenesis

Although WOXs are associated with somatic embryogenesis, their specific functions remain largely unknown. Co-expression analysis can offer potential insights into the roles of their target genes. For example, co-expression and GO enrichment analysis have previously highlighted numerous biological processes, encompassing post-embryonic development, reproductive processes in multicellular organisms, and other processes associated with somatic embryogenesis in plants [62,63]. In *C. arabica*, post-embryonic development, root meristem growth, and multicellular organismal processes were implicated as functions of co-expressed genes of the *CaWOX2* and *CaWOX8* genes (Figure 6), implying their roles in somatic embryogenesis for promoting calluses towards embryogenesis and contributing to the development of roots, shoots, and leaf organs.

Epigenetic modifications play pivotal roles in the signaling cascade that leads to alterations in cell genetic programming, thereby initiating somatic embryo development [64,65]. The regulation of epigenetic mechanisms has recently emerged as a highly promising strategy for improving somatic embryogenesis in plants. Such regulation can be orchestrated through processes such as DNA methylation, chromatin remodeling, and small-RNA-mediated regulation [57,66]. For example, *LEC2* (*Leafy Cotyledon 2*) can be rapidly induced by auxin through chromatin remodeling. Subsequently, activated *LEC2* promotes the expression of *WOX2* and *WOX3*, thereby facilitating somatic embryo formation [64]. In *C. arabica*, the regulation of chromatin organization, histone modification, histone H3-K4 methylation, histone lysine methylation, and other epigenetic processes was implicated as functions of genes co-expressed with the *CaWOX2* and CaWOX8 genes (Figure 6), suggesting that *CaWOX* genes may also regulate somatic embryogenesis by epigenetic means.

*CaWOX13a* and *CaWOX13b* function as negative regulators for embryonic callus formation, and secondary metabolite biosynthetic processes were implicated as functions among the genes that were co-expressed with them (Figure 4, Figure 5 and Figure 6). In cotton, secondary metabolite contents in the embryonic callus were less abundant than in a non-embryonic callus [67]. Phenylpropanoid metabolism may reduce oxidative stress in non-embryonic callus and promote embryogenic capacity [43,68,69]. Here, the phenylpropanoid biosynthesis pathway was represented by *CaWOX13a* and *CaWOX13b* that were highly expressed in explants from the dedifferentiation stage to the primary callus stage (Figure 4, Figure 5 and Figure 6). Taken together, these results demonstrate that CaWOX participates in or regulates somatic embryogenesis through a multitude of biological processes.

### 3.5. CaWOX and Cold Stress

Low temperatures can cause irreversible damage to plants and adversely affect their growth and development. However, many plants enhance their cold tolerance through a process known as cold acclimation (CA) [70]. During cold stress, the expression of cold-regulated (COR) genes is induced, as controlled by various transcription factors (TFs). This induction leads to changes in the physiological and biochemical characteristics of the plant. Among these TFs, C-repeat binding factors (CBFs) are particularly well represented [71]. In *Arabidopsis*, *AtWOX6* (also known as *HOS9*) can enhance cold tolerance by affecting gene activity independent of the CBF pathway [31]. *WOX6* was found to be inducible by auxin, while cold stress can affect auxin transport and intracellular auxin gradients, suggesting potential links among *WOXs*, auxin, and cold-stress responses in the regulation of plant growth and development [72,73]. However, these links are still poorly understood [74]. In *C. arabica*, no orthologs of *AtWOX6* were identified (Figure 1). However, its closest homologs, *CaWOX1c* and *CaWOX1d*, were more highly expressed under both CA and CT conditions (Figure 7), indicating that *CaWOX1c/d* may act in similar roles as *WOX6*. In pineapple plants, most *WOX* genes were decreased in expression by cold treatment, indicating that some *WOXs* may exert negative roles during cold tolerance [33]. For *C. arabica*, one gene (*CaWOX13a*) was more highly expressed in response to both CA and CT, while another gene (*CaWOX13c*) was only more highly expressed in response to CA (Figure 7), indicating that these genes play vital roles in cold-stress responses.

## 4. Materials and Methods

### 4.1. Identification of WOX Genes in Coffea Species

The entire genome sequences of *C. arabica*, *C. canephora*, and *C. eugenioides* were retrieved from the Online Resource for Community Annotation of Eukaryotes (ORCAE, https://bioinformatics.psb.ugent.be/orcae/overview/Coara, Department of Plant Biotechnology and Bioinformatics, Ghent University, Ghent, Belgium, accessed on 16 August 2023), Coffee Genome Hub (http://coffee-genome.org, accessed on 16 August 2023), and NCBI (https://www.ncbi.nlm.nih.gov/genome, National Center for Biotechnology Information, National Library of Medicine, Bethesda, MD USA, accessed on 16 August 2023), respectively [41,42]. The protein sequences of 15 AtWOXs were retrieved from The Arabidopsis Information Resource (TAIR, http://www.arabidopsis.org, Phoenix Bioinformatics Corporation, Newark, CA, USA, accessed on 16 August 2023) [9]. These 15 AtWOX protein sequences were used as queries to perform a BLASTp search (*E*-value < 1 × 10^−5^) against all of the protein sequences of the three coffee plants. Candidate protein sequences of the three Coffea plants identified from BLASTp searches were used as queries to search against the Pfam HMM library (Pfam 34.0) [75,76] using a Hidden Markov Model (HMM) profile (version 3.1.b2). Search hits with a Homeobox domain (PF00046) were identified and considered WOX family candidates based on an *E*-value cutoff of 0.001. The amino acid sequences of the WOX candidates were analyzed to determine the presence of three unique motifs (FYWFQNH, FYWFQNR, and YNWFQNR) previously defined for WOXs [6]. Candidates without unique motifs were not considered further. All *Coffea arabica* WUSCHEL-related homeobox (CaWOX) genes were identified based on their best hit among Arabidopsis WOX proteins.

### 4.2. Phylogenetic and Bioinformatic Analysis of CaWOXs

Multiple alignments of the WOX protein sequences from the three coffee plants and *Arabidopsis* were performed using the MUSCLE program implemented in MEGA6 with default parameters [77]. Unrooted phylogenetic trees were constructed using MEGA6 with neighbor-joining methods and analysis parameters including 1000 bootstrap replicates, and the Jones–Taylor–Thornton (JTT) substitution model [78]. The isoelectric points (PIs) and molecular weights (MWs) of the CaWOXs were analyzed using the ProtParam tool (Expasy, the Swiss Bioinformatics Resource Portal, https://web.expasy.org/protparam, accessed on 16 August 2023) [79]. The exon–intron gene structures of *CaWOXs* were represented using the Gene Structure Display Server (GSDS, version 2.0, http://gsds.gao-lab.org, accessed on 16 August 2023) based on the genomic General Feature Format (GFF) annotation file [80]. Conserved motifs were identified using the MEME software program (version 5.5.2, http://meme-suite.org, accessed on 16 August 2023) [81] based on the default settings, and the results were analyzed using TBtools [82]. The identified motifs were annotated using comparisons against the InterPro (https://www.ebi.ac.uk/interpro, accessed on 16 August 2023) [83] and NCBI databases.

### 4.3. Cold Treatment, Electrolyte Leakage Test, and Tissue Culture

The 1-year-old seedlings of *C. arabica* (Catimor, CIFC7963) were subjected to cold treatment. Cold treatments were carried out as described previously [84]. For the control group (CK), the plants were subjected to a temperature of 24 °C during the day and 20 °C at night for 7 days in a growth chamber. This was followed by another 7 days at 13 °C during the day and 8 °C at night to induce cold acclimation (CA). Subsequently, the plants were exposed to 4 °C both during the day and at night for 3 days (CT). Throughout the cold treatment, the environmental conditions were maintained at a humidity of 60%, a luminosity of 600–650 μmol m^−2^s^−1^, and a photoperiod of 16 h light and 8 h dark. At the end of each treatment period, five plants were selected, and the two most recently matured pairs of leaves were collected from the top of the plants. These leaves were immediately frozen in liquid nitrogen and stored at −80 °C for later analysis. The entire cold treatment process was repeated three times independently with different sets of plants.

Immediately after cold treatment, electrolyte leakage was measured in cold-treated and control plants as previously described [85], with some modifications. Briefly, five leaf discs were randomly picked from a mix of discs (10 discs per plant from the 5 most recently fully expanded leaves that were mixed together). These discs were placed in a glass tube containing 10 mL of distilled water. The samples were then incubated on an orbital shaker at 150 rpm for 30 min at room temperature. Then, initial conductivity (I) was measured using a CON110 conductivity meter (Oakton Ins., Vernon Hills, USA). Next, the leaf discs were boiled in water for 10 min and cooled to room temperature, and the final conductivity (F) was measured. The relative electrolyte leakage was calculated using the following formula: I/F×100.

For tissue culture, the second pair of fully expanded leaves (from top to bottom) was collected from two-year-old coffee seedlings (*C. arabica* L. cv. ‘Catimor CIFC 7963’). These leaves were used as explants. The coffee seedlings were grown under greenhouse conditions at Yunnan University in the spring of 2021, as follows: 16 h light/8 h dark, 60% humidity, 600–650 µmol m^−2^ s^−1^ light intensity, and 24/20 °C (day/night). The explant leaves were sterilized with 70% alcohol and 1% sodium hypochlorite. After sterilization, the explants were washed 4 or 5 times with distilled water and dissected into 0.5 cm^2^ blocks. The blocks were then placed on half-strength Murashige and Skoog (MS) solid medium supplemented with 1 mg/L 6-Benzylaminopurine (6-BA) and 2 mg/L 2,4-Dichlorophenoxyacetic acid (2,4-D) for callus induction. After 2 months of cultivation, the induced callus was transferred to embryo induction medium, which contained 0.5 mg/L 1-Naphthaleneacetic acid (NAA), 5 mg/L 6-BA, and 4 mg/L AgNO_3_. After 6, 7, and 9 months of cultivation, the compact primary callus, embryogenic callus, and globular embryos were collected for further analysis and frozen immediately in liquid nitrogen, followed by storage at −80 °C until subsequent use.

### 4.4. Expression Analysis of CaWOX Genes Within RNA-Seq Data

To analyze *CaWOX* expression among tissues, publicly available RNA-seq data from 14 *C*. *arabica* organs or tissues were retrieved from the NCBI database via the BioProject accessions PRJNA339585, PRJNA305756, and PRJNA5546 [10,86]. Data from tissues comprising roots, stems, leaves, meristems, flower buds, flowers, fruit perisperm (5 samples), and 10-day imbibed seeds (3 samples) were included. The sequencing reads were mapped to the new published reference genome (“ET-39”) using the HISAT2 (version 2.1.0) software program [41,87]. Gene expression levels were re-calculated using the transcripts per million (TPM) metric, and heatmaps for *CaWOX* expression profiles were generated using the TBtools software program (version 1.0987663) [83].

The expression of *CaWOX* involved in the somatic embryogenesis (SE) developmental process was recalculated from a previous study (BioProject PRJNA744419) using the TPM metric [43]. Thirteen sampling stages were selected to encompass the entire somatic embryogenesis (SE) process, ranging from leaf explant initiation to the torpedo-shaped embryo development, as demonstrated in previous studies [43,88]. Leaves collected from greenhouse plants (designated L1), explants undergoing dedifferentiation at 1 (D1), 2 (D2), and 5 weeks (D3), compact primary callus obtained 3 months post-induction (C1), embryogenic callus harvested 7 months post-induction (C2), established cell clusters after 4 months in liquid proliferation medium (C3), pro-embryogenic masses at various stages in redifferentiation medium 1 week after auxin withdrawal (R1), 24 h (R2), 72 h (R3), and 10 days after reducing cell density (R4)], and globular embryos developed after 3 weeks of culture (E1) were sampled.

The TPM values of *CaWOX* genes were also recalculated after drought, elevated air CO_2_, nitrogen stress, and elevated temperature treatments, as described in previous studies (BioProjects PRJNA282394, PRJNA606444, PRJEB15539, and PRJNA609253) [44,45,89,90]. The drought stress experiment was conducted using two contrasting *Coffea arabica* cultivars: Rubi MG1192 (Rubi, drought-susceptible) and IAPAR59 (I59, drought-tolerant). Samples of the shoot apices were collected for RNA-seq analysis [44]. The treatment involving elevated atmospheric CO_2_ was conducted using the 1.5-year-old Icatu variety, with CO_2_ concentrations set at 380 μmol mol^−1^ (ambient CO_2_ or aCO_2_) and 700 μmol mol^−1^ (elevated CO_2_ or eCO_2_). The newly matured leaves from plagiotropic and orthotropic branches were collected for RNA-seq [90]. In nitrogen-stress experiments, the lateral roots of the IAPAR59 variety were exposed to nitrogen-free conditions for 0, 1, and 10 days [89]. Elevated temperature treatment was carried out using two *Coffea arabica* genotypes, cvs. Acauã and Catuaí IAC 144, which have been suggested to differ in heat tolerance [45].

### 4.5. Co-Expression and GO Enrichment Analysis

Candidate *CaWOXs* were used as target genes for genome-wide co-expression analysis to identify genes based on the expression profiling of *C. arabica* RNA-seq data during somatic embryogenesis [43]. A cutoff threshold of <−0.9 or >0.9 was used for Pearson correlation coefficient (PCC) analysis. Gene Ontology (GO) enrichment analysis was performed using agriGO with a false *discovery rate* (FDR) value below 0.05 [91], and the top 20 GO enrichment items were visualized using R packages.

### 4.6. RNA Isolation, cDNA Synthesis, and Quantitative Real-Time PCR

Following the manufacturer’s instructions, total RNA was isolated from homogenized samples using the RNAiso Plus Reagent (Takara Biomedical Technology Co., Ltd., Beijing, China). First-strand cDNA was synthesized using the PrimeScript™ RT Reagent Kit (Takara Biomedical Technology Co., Ltd., Beijing, China) using 1 μg of total RNA. The synthesized cDNA was diluted to 10 ng/μL for PCR. Quantitative real-time PCR (qRT-PCR) was conducted using the QuantStudio™ 7 Flex Real-Time PCR System (Applied Biosystems^®^, Foster City, CA, USA), with the following reaction system specifications: a total volume of 20 µL, consisting of 2 µL of cDNA, 1.0 µL of each primer at a 10 µM concentration, 10 µL of SYBR^®^ Premix Ex Taq™ II (Tli RNaseH Plus) (TAKARA BIO INC., Shiga, Japan), and 6 µL of distilled water. The thermal cycling protocol for PCR involved an initial denaturation step at 95 °C for 30 s, followed by 40 cycles of denaturation at 95 °C for 5 s and extension at 56 °C for 30 s. The PCR results were analyzed using the 2^−∆∆C^_T_ method, and all primer sequences used are listed in Table S6.

## 5. Conclusions

In this study, genome-wide identification of *WOX* genes in three *Coffea* species was conducted. A total of 45 *WOX* genes were identified classified into three clades, consistent with previous studies. During somatic embryogenesis in *C. arabica*, only half of the *WOX* genes were actively expressed, with the expression patterns indicating that different *WOX* gene family members might function in different somatic embryogenesis stages of *C. arabica*. *CaWOX2s*, *CaWOX8s*, *CaWOX13a*, and *CaWOX13b* emerged as potential key genes that could play important roles in somatic embryogenesis. Co-expression analysis further suggested various specific biological processes that *CaWOXs* may be involved in during somatic embryogenesis. Additionally, four *CaWOX* genes were identified that responded to cold stress via altered expression levels. The comprehensive analysis of the *CaWOX* gene family in this study and its roles in both *C. arabica* somatic embryogenesis and stress response offers valuable insights to promote future functional experimental studies.

## Figures and Tables

**Table 1:** Gene information for *WOX* from three coffee plants. bp, base pair; a.a., amino acid; MW, molecular weight; Kda, kilodalton; pI, isoelectric point.

Gene Name	Gene ID	Chromosome Location	gDNA Length (bp)	Protein Length (a.a.)	MW (KDa)	Isoelectric Point (pI)	Best BLAST Hit Within *Arabidopsis*		
							*At*_ID	*At*_Name	*E*-Value
*CaWOX3a*	*Cara001g022170*	Chr 01	1199	216	24.88	9.35	*AT2G28610.1*	*AtWOX3*	5.50 × 10^−46^
*CaWOX2a*	*Cara001g022960*	Chr 01	1360	261	29.45	8.79	*AT5G59340.1*	*AtWOX2*	9.46 × 10^−52^
*CaWOX3b*	*Cara002g013730*	Chr 02	1197	216	24.87	9.35	*AT2G28610.1*	*AtWOX3*	3.45 × 10^−43^
*CaWOX3c*	*Cara002g013760*	Chr 02	1197	216	24.87	9.35	*AT2G28610.1*	*AtWOX3*	3.45 × 10^−43^
*CaWOX2b*	*Cara002g014580*	Chr 02	1369	262	29.44	9.05	*AT5G59340.1*	*AtWOX2*	2.33 × 10^−49^
*CaWOX8b*	*Cara003g039800*	Chr 03	2097	407	45.00	8.75	*AT5G45980.1*	*AtWOX8*	1.39 × 10^−78^
*CaWOX5a*	*Cara003g048620*	Chr 03	656	174	19.70	8.76	*AT3G11260.1*	*AtWOX5*	2.89 × 10^−60^
*CaWOX5b*	*Cara004g007900*	Chr 04	666	174	19.70	8.76	*AT3G11260.1*	*AtWOX5*	2.89 × 10^−60^
*CaWOX8a*	*Cara004g016390*	Chr 04	2092	407	44.89	8.55	*AT5G45980.1*	*AtWOX8*	5.16 × 10^−79^
*CaWOX1a*	*Cara007g007070*	Chr 07	2160	362	40.90	6.18	*AT3G18010.1*	*AtWOX1*	5.26 × 10^−60^
*CaWOX13d*	*Cara007g012080*	Chr 07	741	247	28.26	5.43	*AT4G35550.1*	*AtWOX13*	2.86 × 10^−49^
*CaWOX13c*	*Cara008g014990*	Chr 08	741	247	28.26	5.54	*AT4G35550.1*	*AtWOX13*	2.31 × 10^−49^
*CaWOX1b*	*Cara008g019840*	Chr 08	2164	362	40.97	6.53	*AT3G18010.1*	*AtWOX1*	3.10 × 10^−59^
*CaWOX11a*	*Cara011g029950*	Chr 11	2412	257	27.90	5.61	*AT3G03660.1*	*AtWOX11*	2.40 × 10^−63^
*CaWOX11b*	*Cara012g008700*	Chr 12	2410	257	27.90	5.61	*AT3G03660.1*	*AtWOX11*	2.40 × 10^−63^
*CaWUSb*	*Cara013g011100*	Chr 13	1611	286	32.12	6.45	*AT2G17950.1*	*AtWUS*	2.41 × 10^−46^
*CaWOX13b*	*Cara013g012460*	Chr 13	3119	280	31.00	5.56	*AT4G35550.1*	*AtWOX13*	9.66 × 10^−87^
*CaWOX13a*	*Cara014g017290*	Chr 14	3604	278	30.89	5.56	*AT4G35550.1*	*AtWOX13*	9.35 × 10^−89^
*CaWUSa*	*Cara014g018710*	Chr 14	1612	286	32.14	6.45	*AT2G17950.1*	*AtWUS*	2.03 × 10^−46^
*CaWOX4a*	*Cara019g006470*	Chr 19	1122	222	25.44	9.51	*AT1G46480.1*	*AtWOX4*	1.00 × 10^−76^
*CaWOX4b*	*Cara020g021370*	Chr 20	1078	222	25.47	9.51	*AT1G46480.1*	*AtWOX4*	1.36 × 10^−76^
*CaWOX1d*	*Cara021g016240*	Chr 21	1526	337	38.23	7.15	*AT3G18010.1*	*AtWOX1*	1.03 × 10^−31^
*CaWOX1c*	*Cara022g010550*	Chr 22	1526	337	38.26	6.84	*AT3G18010.1*	*AtWOX1*	9.18 × 10^−32^
*CcWOX11*	*Cc00g05100*	Chr un	2503	251	27.45	5.84	*AT3G03660.1*	*AtWOX11*	9.05 × 10^−65^
*CcWOX3a*	*Cc00g26230*	Chr un	1198	215	24.93	9.51	*AT2G28610.1*	*AtWOX3*	2.71 × 10^−45^
*CcWOX3b*	*Cc01g11960*	Chr 01	1191	215	24.87	9.57	*AT2G28610.1*	*AtWOX3*	8.83 × 10^−42^
*CcWOX2*	*Cc01g12690*	Chr 01	1359	237	26.88	8.93	*AT5G59340.1*	*AtWOX2*	3.43 × 10^−52^
*CcWOX5*	*Cc02g06840*	Chr 02	666	173	19.70	8.76	*AT3G11260.1*	*AtWOX5*	2.79 × 10^−60^
*CcWOX8*	*Cc02g14220*	Chr 02	2087	406	45.00	8.75	*AT5G45980.1*	*AtWOX8*	1.35 × 10^−78^
*CcWOX1a*	*Cc04g06330*	Chr 04	3252	361	40.97	6.05	*AT3G18010.1*	*AtWOX1*	3.52 × 10^−60^
*CcWOX13b*	*Cc04g10680*	Chr 04	740	246	28.26	5.43	*AT4G35550.1*	*AtWOX13*	2.77 × 10^−49^
*CcWUS*	*Cc07g10660*	Chr 07	1609	285	32.09	6.45	*AT2G17950.1*	*AtWUS*	2.66 × 10^−46^
*CcWOX13a*	*Cc07g11890*	Chr 07	3819	279	31.00	5.56	*AT4G35550.1*	*AtWOX13*	9.32 × 10^−87^
*CcWOX4*	*Cc10g04700*	Chr 10	2384	221	25.47	9.51	*AT1G46480.1*	*AtWOX4*	1.31 × 10^−76^
*CcWOX1b*	*Cc11g08460*	Chr 11	1525	336	38.23	7.15	*AT3G18010.1*	*AtWOX1*	1.01 × 10^−31^
*CeWOX2*	*LOC113774651*	Chr 1	1429	260	29.35	9.05	*AT5G59340*	*AtWOX2*	1.22 × 10^−50^
*CeWOX3*	*LOC113774781*	Chr 1	1196	215	24.87	9.35	*AT2G28610*	*AtWOX3*	3.34 × 10^−43^
*CeWOX4*	*LOC113749539*	Chr 10	1482	221	25.47	9.51	*AT1G46480*	*AtWOX4*	1.31 × 10^−76^
*CeWOX1a*	*LOC113753065*	Chr 11	2362	336	38.27	7.6	*AT3G18010*	*AtWOX1*	9.75 × 10^−32^
*CeWOX5*	*LOC113755443*	Chr 2	663	173	19.70	8.76	*AT3G11260*	*AtWOX5*	2.79 × 10^−60^
*CeWOX8*	*LOC113764000*	Chr 2	2406	392	43.19	8.25	*AT5G45980*	*AtWOX8*	1.04 × 10^−78^
*CeWOX1b*	*LOC113768485*	Chr 4	3006	361	40.97	6.53	*AT3G18010*	*AtWOX1*	3.01 × 10^−59^
*CeWOX13a*	*LOC113769015*	Chr 4	797	265	30.71	5.66	*AT4G35550*	*AtWOX13*	4.76 × 10^−49^
*CeWOX13b*	*LOC113778647*	Chr 7	3557	271	30.26	5.56	*AT4G35550*	*AtWOX13*	2.39 × 10^−86^
*CeWOX11*	*LOC113758086*	Chr un	2155	251	27.45	5.84	*AT3G03660*	*AtWOX11*	9.05 × 10^−65^

## Data Availability

Data are contained within the article and Supplementary Materials.

## Supplementary Materials

The following supporting information can be downloaded at https://www.mdpi.com/article/10.3390/ijms252313031/s1.
