# Supplementary material for: The Identification and Characterization of WOX Family Genes in Coffea arabica Reveals Their Potential Roles in Somatic Embryogenesis and the Cold-Stress Response
Source: Int J Mol Sci. 2024 Dec 4;25(23):13031. doi: 10.3390/ijms252313031 (PMC11641659; doi:10.3390/ijms252313031)
Supplement: Supplementary file 1 [file ijms-25-13031-s001.zip › Table S1.pdf]

Supplementary Table S1. Conserved motifs identified in the WOX proteins.

Motif No.	Motif consensus sequence	*E*-value	Motif Width	No. of WOX proteins	Motif annotation
1	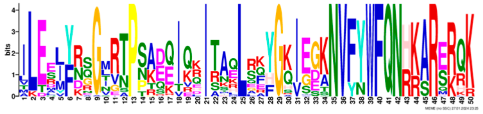	3.4e-2120	50	60	homeodomain super family
2	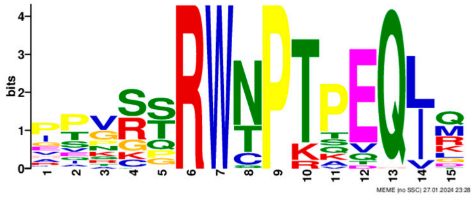	1.5e-436	15	60	—
3	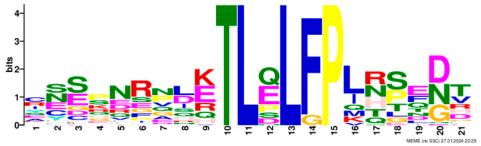	2.0e-230	21	40	—
4	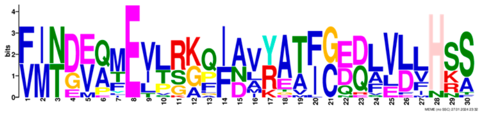	6.6e-324	30	23	—
5	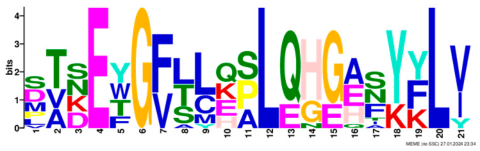	2.7e-127	21	16	—

6	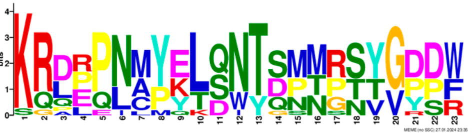	3.5e-099	23	14	—
7	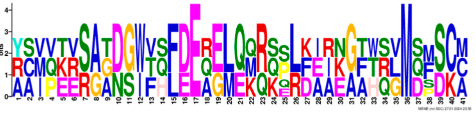	8.7e-185	41	12	—
8	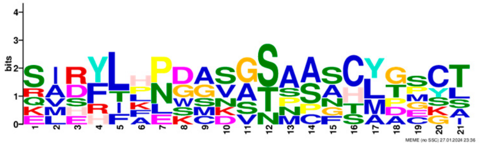	2.4e-109	26	21	—
9	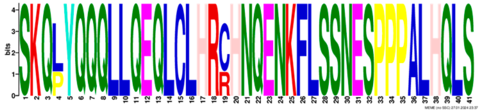	1.6e-140	41	6	—
10	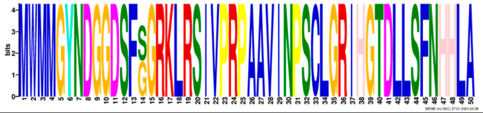	1.5e-110	50	4	—
